# Systemic immune responses in patients with early localized or early disseminated *Borrelia afzelii* lyme borreliosis

**DOI:** 10.1002/iid3.398

**Published:** 2020-12-31

**Authors:** Tjaša Cerar Kišek, Rok Blagus, Eva Ružić‐Sabljić, Stefan Collinet‐Adler, Fajko F. Bajrović, Daša Stupica

**Affiliations:** ^1^ Faculty of Medicine Ljubljana Institute of Microbiology and Immunology Ljubljana Slovenia; ^2^ Faculty of Medicine Ljubljana Institute for Biostatistics and Medical Informatics Ljubljana Slovenia; ^3^ Faculty of Sports University of Ljubljana Ljubljana Slovenia; ^4^ Department of Infectious Diseases Methodist Hospital Saint Louis Park Minnesota USA; ^5^ Department of Neurology University Medical Center Ljubljana Ljubljana Slovenia; ^6^ Faculty of Medicine Ljubljana Ljubljana Slovenia; ^7^ Department of Infectious Diseases University Medical Center Ljubljana Ljubljana Slovenia

**Keywords:** borrelial dissemination, inflammatory mediators, lyme borreliosis, lyme borreliosis‐associated symptoms

## Abstract

**Introduction:**

The role of host immune responses in the pathogenesis of borrelial dissemination in early Lyme borreliosis (LB) in the form of multiple erythema migrans (MEM) or LB‐associated symptoms is incompletely understood.

**Methods:**

In this study, fifteen cytokine or chemokine levels, representative of innate, Th1, and Th17 immune responses, were assessed using a bead‐based Luminex multiplex assay in acute sera from 76 adult patients with skin culture‐positive *Borrelia afzelii* solitary erythema migrans (SEM) and 58 patients with MEM at a single‐center university hospital. Differences between the groups were tested by modeling each cytokine or chemokine concentration by means of left‐censored regression using the classic Tobit model.

**Results:**

Mean serum cytokine or chemokine levels were low. When taking into account the proportion of patients with cytokine or chemokine concentrations below the lowest detectable limit, only levels of CXCL10 (*p = *.03) and CCL19 (*p* = .02), representatives of the Th1 immune response, differed between patients with SEM and those with MEM; however, the differences did not reach statistical significance when adjusted for multiple comparisons. In addition, we did not find differences in systemic inflammatory responses when comparing patients with and those without LB‐associated constitutional symptoms.

**Conclusion:**

No significant differences in systemic immune responses represented by selected cytokines or chemokines in serum samples of patients with EM infected with *B. afzelii* suggest that systemic mediators are not pivotal in the pathogenesis of dissemination of early infection in the form of MEM or LB‐associated symptoms. Localized immune responses in the skin or other pathogenetic mechanisms may be more important in this regard.

**Abbreviations**
EM: erythema migrans; B, *Borrelia*
LB: Lyme borreliosis
MEM: multiple erythema migrans
SEM: solitary erythema migrans

## 1 INTRODUCTION

Erythema migrans (EM) is the most common clinical manifestation of early Lyme borreliosis (LB), which is caused by *Borrelia burgdorferi* sensu lato (s.l.).^1^ Solitary EM (SEM) results from inflammation generated by local immune responses to borreliae inoculated into the skin during tick feeding, while multiple EM (MEM) results from inflammation at distant skin sites developing several days after onset of the primary skin lesion due to hematogenous dissemination of bacteria from the initial inoculation site.^2^ Hematogenous dissemination may arise from infection with more invasive strains of borreliae,^1^, ^2^, ^3^, ^4^ failure of patients to mount an adequate immune response,^5^ the immunosuppressant activities of tick salivary proteins,^6^ or a combination of these factors.

In comparison with *B. burgdorferi* sensu stricto (further reported as *B. burgdorferi*) in the U.S., the most prevalent borrelial species in Europe, *B. afzelii*, causes EM which has slower progression of local inflammatory signs, presents less often with disseminated disease as evidenced by spirochetemia or presence of MEM, is less often accompanied by LB‐associated constitutional symptoms, and induces weaker immune responses, as indicated by lower levels of both innate and adaptive immune response mediators detected in skin and serum samples from patients with EM.^2^, ^7^, ^8^, ^9^, ^10^, ^11^

In an effort to understand the role of immune responses in the pathogenesis of dissemination of borrelial infection, several previous studies examined cytokine or chemokine profiles in patients with SEM and MEM, but their results are inconclusive,^5^, ^10^, ^12^, ^13^ and to our knowledge no such comparisons have been performed in *B. afzelii* EM.

The aim of this study was to further investigate the association between systemic innate, Th1, and Th17 immune responses and dissemination of early borrelial infection by comparing these responses in patients with skin culture‐positive *B. afzelii* localized or disseminated early LB manifested as SEM or MEM. We also explored the association between markers of serum immune responses and LB‐associated symptoms.

## 2 MATERIALS AND METHODS

### 2.1 Setting and participants

Between June 2006 and October 2015, 1020 patients with SEM and 200 patients with MEM were enrolled prospectively in five clinical trials at the University Medical Center Ljubljana, Slovenia.^11^, ^14^, ^15^, ^16^, ^17^ We selected 58 patients with culture‐positive *B. afzelii* MEM, who were 18 years of age, had no history of LB in the past, had attended the 2‐ and 12‐month follow‐up visits, and for whom sufficient volumes of sera for analysis were available. Because of limited financial resources, 76 age and sex‐matched patients with solitary *B. afzelii* EM were randomly selected from the group of 1020 patients, using the same inclusion criteria.

SEM was defined as an expanding erythema, with or without central clearing, developing days to weeks after a tick bite or after exposure to ticks in an LB endemic region. For a reliable diagnosis, the erythema must have a diameter of at least 5 cm. If the diameter was smaller, a history of tick bite, a delay in appearance of at least two days, and an expanding erythema at the site of the bite were required.^18^ MEM was defined as the presence of two or more erythemas, at least one of which had to fulfill the size criterion for SEM.^1^ At enrollment, patients were prescribed antibiotics in accordance with treatment guidelines.^19^

### 2.2 Evaluation of patients

At baseline and at follow‐up (14 days and 2, 6, and 12 months), the patients were physically examined and their medical histories were collected. Patients were also asked, without prompting, an open question about any health‐related symptoms that had newly developed or worsened since the onset of the EM. If these symptoms had no other medical explanation, they were considered as LB‐associated constitutional symptoms at enrollment or post‐LB symptoms at follow‐up.

Complete response to treatment was defined as a return to pre‐LB health status. Incomplete recovery was defined as the presence of post‐LB symptoms (partial response), or as the occurrence of new objective signs of LB and/or persistence of borreliae as detected by culture of a re‐biopsied skin sample, and/or persistence of EM that could still be seen in daylight and at room temperature at 2 months posttreatment (treatment failure).

### 2.3 Laboratory analyses

After collection, serum samples were immediately stored at ‒20°C until used in this study. To maintain integrity, the samples did not undergo freeze‐thaw before the cytokine or chemokine analysis. Based on results of previous studies,^10^, ^20^, ^21^, ^22^ bead‐based Luminex (EMD‐Millipore) multiplex assays were used to analyze selected cytokines or chemokines representative of innate (interleukin‐1β [IL‐1β], IL‐6, IL‐8, IL‐10, tumor necrossis factor‐α [TNF‐α], CCL2), adaptive Th1 (IL‐12P70, IFNγ, CXCL9, CXCL10, CCL19), and adaptive Th17 (IL‐17F, IL‐21, IL‐23, IL‐27) immune responses in serum samples from patients at enrollment. To minimize inter‐assay variation, all measurements in a single panel were performed on the same day in one complete experiment, following the manufacturer's instructions.

Serologic data were obtained using indirect chemiluminescence immunoassays for immunoglobulin M (IgM) antibodies to OspC and VlsE and immunoglobulin G (IgG) antibodies to VlsE borrelial antigens (Liaison). Results were interpreted according to the manufacturers' instructions. Modified Kelly–Pettenkofer medium was used for cultivation of *B. burgdorferi* s.l. from skin samples as described elsewhere.^23^ Isolates were identified to species or strain level using MluI restriction of genomic DNA (MluI‐length restriction fragment patterns) or by MseI restriction of rrf (5S)–rrl (23S) intergenic spacer amplicons (MseI‐restriction fragment‐length polymorphism).^23^, ^24^

### 2.4 Statistical analyses

Data for cytokine or hemokine concentrations are presented as medians and interquartile range (IQR) and, considering only the values above the detection limit, as means and standard deviations; the proportion of values above the detection limit is also reported. Mann–Whitney test was used for the between groups comparisons. To account for the left‐censoring of cytokine or chemokine concentrations (values below the detection limit), the SEM and MEM groups were compared as follows. Data were first log transformed (using natural logarithms) to ensure that the normality assumption was met. The differences between the groups were tested by modeling each cytokine or chemokine concentration by censored regression using the classic Tobit model where the group variable is modeled as an independent variable. The model parameters were estimated using the method of maximum likelihood, assuming the normal distribution of the error term.^25^ Multivariate analysis, adjusting for age and sex, was also performed. Censored regression was used also for assessing the association between cytokine or chemokine concentrations and duration, diameter and presence/absence of LB‐associated symptoms (considered as independent variables in separate models) adjusting the analysis also for sex, age, and dissemination (SEM vs. MEM). The association between cytokine or chemokine concentrations and specific immune responses to borrelial antigens was estimated with Spearman's rho rank based correlation coefficient, testing the correlation using the algorithm AS89.^26^ To account for multiple comparisons, *p* values were adjusted using the Benjamini–Hochberg method to control the false discovery rate at 5% (an adjusted *p*< .05 was considered statistically significant). R language for statistical computing (R version 3.5.1) was used for the analysis.^27^ The censored regression was fitted using the censReg package in R. The algorithm AS89 was implemented using the R package pspearman.

## 3 RESULTS

Basic demographic, clinical, and serologic information on the acute disease and posttreatment outcome in the 76 patients with SEM and 58 patients with MEM is shown in Table 1.

**Table 1:** Characteristics of patients with solitary erythema migrans (SEM) and multiple erythema migrans (MEM) at enrollment and follow‐up

**Characteristic**	**SEM (*n* = 76)**	**MEM (*n* = 58)**	***p***
Age, years	44.5 (36.8–59.0)	45.0 (35.3–53.0)	.21
Male sex	38 (50%)	27 (46.6%)	.83
Comorbidities^a^	31 (40.8%)	24 (41.4%)	1.0
Days since EM first observed	12.5 (5–31)	7 (6–15.5)	.09
Diameter of primary EM, cm	18.5 (11.8–29.3)	12.5 (8.3–20)	<.001
LB‐associated constitutional symptoms at enrollment^b^	18 (23.7)	24 (41.4)	.046
Fatigue	9 (11.8)	13 (22.4)	.16
Arthralgia	7 (9.2)	11 (18.9)	.17
Headache	8 (10.5)	12 (20.7)	.16
Myalgia	5 (6.6)	5 (8.6)	.75
Seropositive^c^	59 (77.6)	55 (94.8%)	.01
Incomplete recovery at			
14 Days post‐enrollment	17/76 (22.4)	20/55 (36.4)	.12
2 Months post‐enrollment	11/76 (14.5%)	10/58 (17.2%)	.84
6 Months post‐enrollment	3/74 (4.1%)	6/56 (10.7%)	.17
12 Months post‐enrollment	4/76 (5.3%)	3/58 (5.2%)	1.0

*Note:* Data are median (IQR), *n* (%), or n/*n* (%).

Abbreviations: EM, erythema migran; IQR, interquartile range; LB, lyme borreliosis.

a Patients with underlying chronic illness.

b Patients who reported LB‐associated constitutional symptoms at enrollment. Some patients had more than one symptom.

c Positive test result for immunoglobulin M and/or immunoglobulin G to *B. burgdorferi* sensu lato at enrollment.

### 3.1 Clinical characteristics according to dissemination

Patients from the SEM and MEM group did not differ in regard to age, sex distribution, chronic comorbidities and duration of EM since first observed. At enrollment, patients with SEM compared with patients with MEM had larger EM, reported LB‐associated constitutional symptoms less often (18/76, 23.7% vs. 24/58, 41.4%; *p* < .046), and were less likely seropositive for borreliae. The proportion of patients with incomplete recovery, represented predominantly by the presence of post‐LB symptoms, steadily decreased and was comparable between the two groups during follow‐up.

### 3.2 Inflammatory responses according to dissemination

**Figure 1:**
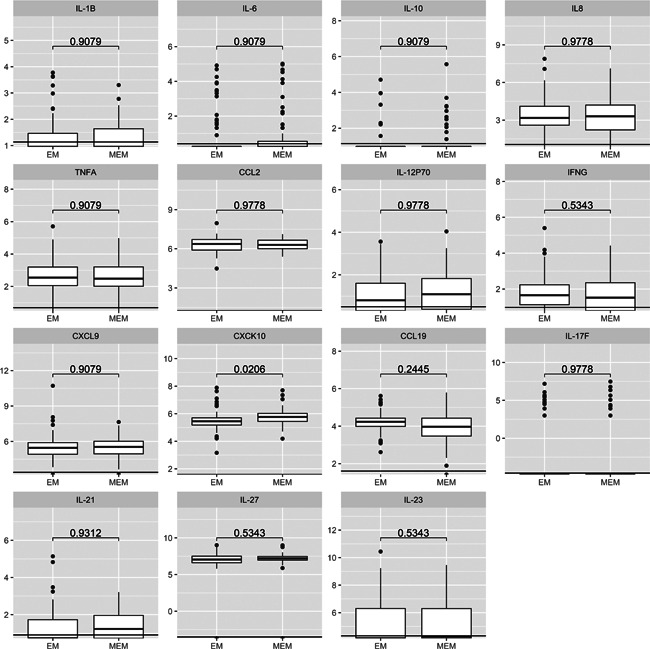
Cytokine or chemokine Levels (pg/ml) in serum from 76 patients with solitary erythema migrans (SEM) and 58 patients with multiple erythema migrans (MEM). Data are presented on the log scale. Vertical lines show lower limits of detection. *p* Values were obtained using Mann–Whitney test and adjusted for multiple comparisons using the Benjamini–Hochberg method

To investigate whether disseminated disease as defined by the presence of MEM, was associated with systemic inflammation, we compared results of the multiplex cytokine or chemokine analysis on serum samples between patients with SEM and those with MEM. Antibiotic therapy was started a median of 12.5 (IQR: 5‒31) days and 7 (IQR: 6‒15.5) days after erythema was first observed in patients with SEM and in those with MEM, respectively. Overall, median serum cytokine levels were low and the proportion of patients with detectable levels of each of the 15 cytokines or chemokines tested ranged from 9.2% to 100% (Table 2 and Figure 1). When taking into account the proportion of patients with cytokine/chemokine concentrations below the lowest detectable limit, mediator concentrations differed significantly between patients with SEM and those with MEM for only two of the mediators tested, CXCL10 and CCL19, both representing Th1 inflammatory response. However, when adjusting the analysis for multiple comparisons, the difference between the two patient groups did not reach significance for any of the mediators tested (Table 2 and Figure 1).

**Table 2:** Cytokine or chemokine levels (pg/ml) in serum from 76 patients with SEM and 58 patients with MEM

**Cytokine/chemokine**	**SEM** a **(*n* = 76)**	**MEM** a **(*n* = 58)**	**Estimate (95% CI)** b	***p***	***p* Adj** d
			**Adjusted estimate (95% CI)** c		
Innate					
IL‐1β	35.5	34.5			
	10.78 ± 11.67	8.38 ± 5.53	−0.04 (−0.56, 0.48)	.88	1.00
	3.11 (3.11‒4.33)	3.11 (3.11‒5.16)	−0.05 (−0.58, 0.47)	.86	.88
IL‐6	21.1	25.9			
	35.38 ± 40.04	54.86 ± 58.77	0.67 (−1.10, 2.45)	.46	.86
	1.51 (1.51‒1.51)	1.51 (1.51‒1.44)	0.57 (−1.16, 2.29)	.52	.73
IL‐10	9.2	19.0			
	38.09 ± 37.71	38.66 ± 75.09	1.31 (−0.47, 3.10)	.15	.56
	3.13 (3.13‒3.13)	3.13 (3.13‒3.13)	1.47 (−0.35, 3.28)	.11	.53
IL‐8	98.7	96.5			
	107.00 ± 336.57	91.37 ± 188.64	−0.10 (−0.57, 0.37)	.68	.90
	23.92 (13.35‒60.63)	27.31 (9.27‒66.52)	−0.10 (−0.57, 0.38)	.69	.79
TNF‐α	98.7	96.5			
	22.77 ± 37.77	25.16 ± 30.13	0.08 (−0.26, 0.43)	.63	.90
	12.75 (7.79‒124.5)	11.96 (7.46‒24.72)	0.13 (−0.21, 0.47)	.44	.73
CCL2	100.0	100.0			
	635.49 ± 377.88	601.71 ± 255.95	−0.00 (−0.18, 0.17)	.96	1.00
	582.40 (365.54‒820.97)	542.78 (406.20‒777.90)	−0.01 (−0.16, 0.19)	.88	.88
Th1					
IL‐12P70	59.2	74.1			
	6.98 ± 7.64	7.07 ± 9.03	0.30 (−0.10, 0.71)	.14	.56
	2.24 (1.65‒4.96)	2.97 (0.45‒6.23)	0.30 (−0.10, 0.70)	.14	.53
IFNγ	77.6	58.6			
	14.20 ± 29.89	14.93 ± 16.29	−0.25 (−0.68, 0.18)	.25	.63
	5.21 (3.08‒9.34)	4.57 (2.65‒10.53)	−0.30 (−0.73, 0.13)	.18	.53
CXCL9	98.7	98.3			
	956.91 ± 5178.34	390.72 ± 402.80	0.00 (−0.33, 0.34)	1.00	1.00
	234.86 (136.76‒364.71)	253.53 (142.15‒410.44)	0.09 (−0.23, 0.41)	.59	.73
CXCL10	100.0	100.0			
	332.09 ± 392.28	385.88 ± 337.31	0.25 (0.02, 0.47)	**.03**	.24
	233.67 (177.70‒299.61)	317.88 (231.19‒410.76)	0.28 (−0.06, 0.51)	**.01**	.22
CCL19	100.0	98.3			
	78.68 ± 46.04	72.18 ± 64.01	−0.29 (−0.52, −0.05)	**.02**	.23
	69.04 (53.78‒83.52)	52.81 (32.14‒83.78)	−0.25 (−0.48, 0.02)	**.04**	.26
Th17					
IL‐17F	19.7	17.2			
	250.00 ± 321.49	395.00 ± 562.32	−1.27 (−8.21, 5.68)	.72	.90
	0.01 (0.01‒0.01)	0.01 (0.01‒0.01)	−1.88 (−8.65, 4.89)	.28	.73
IL‐21	47.4	63.8			
	15.52 ± 33.53	7.79 ± 5.11	0.27 (−0.19, 0.73)	.25	.63
	2.48 (2.48‒5.64)	3.42 (2.48‒7.10)	0.26 (−0.21, 0.72)	.22	.59
IL‐27	96.1	98.3			
	1574.25 ± 1373.84	1616.32 ± 1223.94	0.34 (−0.33, 1.01)	.32	.68
	1145.0 (742.5‒1815.0)	1345.0 (1057.5‒1762.5)	0.42 (−0.25, 1.09)	.22	.55
IL‐23	47.4	53.4			
	2757.97 ± 6003.47	1555.69 ± 2946.83	−0.21 (−1.27, 0.84)	.69	.90
	75.14 (75.14‒546.22)	70.45 (75.14‒541.61)	−0.46 (−1.47, 0.56)	.38	.71

Abbreviations: CI, confidence interval; IFN‐γ, interferon γ; IL, interleukin; MEM, multiple erythema migran; SEM, solitary erythema migrans; TNF, tumor necrosis factor.

a Data in the first row are the percentage of samples with detectable levels. Data in the second row are mean ± standard deviation for values above the detection limit. Data in the third row are median and interquartile range.

b Differences between the SEM and MEM groups as estimated using the Tobit regression model.

c Differences between patients with SEM and MEM as estimated using the Tobit regression model, and adjusted for sex and age.

d *p* Value, adjusted for multiple comparisons.

### 3.3 Immune responses according to selected clinical characteristics and borrelial antibodies in serum

Patients with SEM and MEM were pooled together for investigation of the association between inflammatory responses and selected clinical characteristics and specific borrelial antibodies.

Disease duration varied at the time serum samples were collected because patients had different durations of erythema before referral to our outpatient clinic. This provided us with an opportunity to examine the association between inflammatory immune responses and disease duration. However, the associations between cytokine or chemokine levels and the duration of disease were not significant (Data not shown).

Defining duration of disease from time of initial observation of EM is subjective. To assess disease duration more objectively, we also analyzed the association between inflammatory immune responses and diameter of EM (Table 3). The associations between cytokine/chemokine levels and the diameter of EM were also not significant.

**Table 3:** Association between inflammatory immune responses in serum and diameter of erythema in 134 patients with erythema migrans

**Cytokine/chemokine**	**Estimate (95% CI)** a	***p***	***p* Adj** b	**Adjusted estimate (95% CI)** c	***p***	***p* Adj** b
Innate						
IL‐1β	−0.02 (−0.24, 0.20)	.83	.96	−0.03 (−0.26, 0.19)	.77	.79
IL‐6	0.08 (−0.66, 0.83)	.83	.96	0.22 (−0.56, 1.00)	.58	.79
IL‐10	−0.00 (−0.77, 0.77)	1.00	1.00	0.25 (−0.56, 1.06)	.54	.79
IL‐8	−0.11 (−0.31, 0.08)	.26	.79	−0.14 (−0.35, 0.07)	.18	.79
TNF‐α	−0.09 (−0.23, 0.05)	.21	.78	−0.08 (−0.23, 0.06)	.26	.79
CCL2	0.01 (−0.06, 0.09)	.69	.96	0.02 (−0.06, 0.09)	.66	.79
Th1						
IL‐12P70	−0.16 (−0.33, 0.01)	.08	.53	−0.13 (−0.31, 0.05)	.17	.79
IFNγ	−0.02 (−0.19, 0.16)	.87	.96	−0.06 (−0.25, 0.13)	.52	.79
CXCL9	−0.02 (−0.16, 0.12)	.74	.96	−0.02 (−0.16, 0.12)	.78	.79
CXCL10	−0.09 (−0.18, 0.01)	.08	.53	−0.05 (−0.15, 0.04)	.28	.79
CCL19	0.02 (−0.08, 0.11)	.76	.96	−0.03 (−0.13, 0.07)	.61	.79
Th17						
IL‐17F	−1.11 (−4.10, 1.87)	.47	.96	−1.44 (−4.49, 1.61)	.36	.79
IL‐21	−0.16 (−0.35, 0.03)	.11	.53	−0.14 (−0.35, 0.06)	.18	.79
IL‐27	−0.02 (−0.30, 0.26)	.90	.96	0.04 (−0.25, 0.34)	.79	.79
IL‐23	−0.10 (−0.54, 0.38)	.66	.96	−0.18 (−0.63, 0.26)	.41	.79

Abbreviations: CI, confidence interval; IFN‐γ, interferon γ; IL, interleukin; TNF, tumor necrosis factor.

a The association between the diameter of erythema migrans and cytokine/chemokine levels (pg/ml) as estimated using the Tobit regression model expressed for a difference in diameter of 10 cm.

b *P* value, adjusted for multiple comparisons.

c The association between diameter and cytokine/chemokine levels (pg/ml) as estimated using the Tobit regression model, adjusted for sex, age, and dissemination (solitary vs multiple erythema migrans) expressed for a difference in diameter of 10 cm.

Rho rank correlation values indicated potential positive associations between the Th1 mediator CXCL10 and specific borrelial IgM, the Th1 mediator CXCL9 and specific borrelial IgG, as well as IL‐10 and specific borrelial IgG (Table 4). However, none of these associations were statistically significant after adjustment for multiple comparisons.

**Table 4:** Correlation of cytokine or chemokine levels (pg/ml) and borrelial IgM and IgG antibody levels in serum

**Cytokine/chemokine**	**Correlation with borrelial IgM in serum**			**Correlation with borrelial IgG in serum**		
	**Rho** a	**Unadjusted *p*** b	**Adjusted *p*** c	**Rho** a	**Unadjusted *p*** b	**Adjusted *p*** c
Innate						
IL‐1β	0.081	.35	.52	−0.021	.81	.81
IL‐6	0.114	.19	.49	0.109	0.21	.45
IL‐10	0.116	.18	.49	0.249	**<.01**	.06
IL‐8	0.095	.28	.52	−0.142	.11	.41
TNF‐α	0.096	.27	.52	−0.121	.16	.41
CCL2	−0.034	.70	.75	0.092	.29	.55
Th1						
IL‐12P70	0.111	.19	.49	0.030	.73	.81
IFNγ	−0.003	.97	.97	0.049	.57	.78
CXCL9	0.133	.13	.49	0.219	**.01**	.08
CXCL10	0.248	<.01	.06	0.123	.16	.41
CCL19	−0.062	.48	.65	0.083	.34	.57
Th17						
IL‐17F	−0.049	.57	.71	0.027	.75	.81
IL‐21	0.043	.62	.72	−0.032	.71	.81
IL‐27	0.116	.18	.49	0.124	.15	.41
IL‐23	−0.084	.33	.52	0.049	.57	.78

Abbreviations: IgG, immunoglobulin G; IgM, immunoglobulin M; IL, interleukin; INF‐γ, interferon γ; TNF‐α, tumor necrosis factor α.

a Spearman's rho rank‐based correlation; values >0 indicate positive associations.

b The association was tested using Spearman's method.

c Adjusted for multiple comparisons.

**Figure 2:**
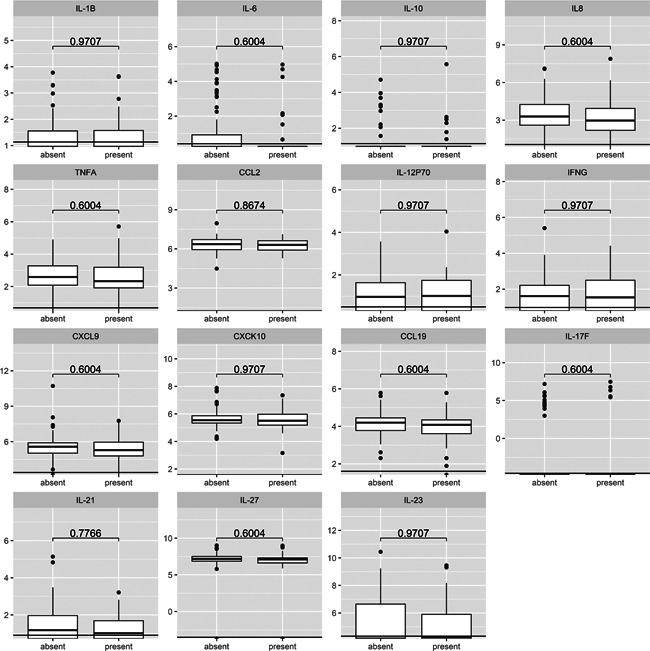
Cytokine or chemokine levels (pg/ml) in serum from 42 patients with Lyme borreliosis‐associated constitutional symptoms and in serum from 92 asymptomatic patients at enrollment. Data are presented on the log scale. Vertical lines show lower limits of detection. *p* Values were obtained using Mann–Whitney test and adjusted for multiple comparisons using the Benjamini–Hochberg method

The levels of 15 mediators tested did not differ significantly between patients who reported LB‐associated constitutional symptoms and those who were asymptomatic at enrollment (Table 5 and Figure 2).

**Table 5:** Cytokine or chemokine levels (pg/ml) in serum from 42 patients with Lyme borreliosis‐associated constitutional symptoms and in serum from 92 asymptomatic patients at enrollment

**Cytokine/chemokine**	**LB‐associated symptoms present (*n* = 42)** a	**LB‐associated symptoms absent (*n* = 92)** a	**Estimate (95% CI)** b	***p***	***p* Adj** d
			**Adjusted estimate (95% CI)** c		
Innate					
IL‐1β	33.3	35.9			
	11.56 ± 11.56	8.96 ± 8.62	0.02 (−0.54, 0.57)	0.95	0.95
	3.11 (3.11‒4.75)	3.11 (3.11‒4.88)	0.01 (−0.55, 0.57)	0.98	0.98
IL‐6	16.7	26.1			
	49.57 ± 58.62	43.42 ± 48.69	−1.25 (−3.26, 0.76)	0.22	0.80
	1.51 (1.51‒2.52)	1.51 (1.51‒1.51)	−1.25 (−3.19, 0.69)	0.21	0.70
IL10	14.3	13.1			
	51.43 ± 103.61	31.94 ± 29.75	0.05 (−1.79, 1.90)	0.95	0.95
	3.13 (3.13‒3.13)	3.13 (3.13‒3.13)	−0.21 (−2.08, 1.66)	0.82	0.88
IL8	97.6	97.8			
	120.79 ± 417.54	90.99 ± 193.99	−0.24 (−0.75, 0.26)	0.34	0.80
	26.81 (13.37‒70.01)	19.42 (8.91‒50.89)	−0.23 (−0.74, 0.28)	0.38	0.70
TNF‐α	95.2	98.9			
	28.29 ± 53.34	21.81 ± 22.00	−0.15 (−0.51, 0.22)	0.42	0.80
	13.31 (8.03‒26.66)	10.33 (6.81‒24.22)	−0.17 (−0.53, 0.19)	0.36	0.70
CCL2	100.00	100.00			
	590.67 ± 272.59	634.65 ± 353.61	−0.05 (−0.23, 0.14)	0.61	0.91
	581.3 (380.61‒821.26)	549.14 (367.96‒755.42)	−0.05 (−0.24, 0.14)	0.61	0.87
Th1					
IL‐12P70	66.7	65.2			
	6.87 ± 10.17	7.09 ± 7.36	−0.02 (−0.45, 0.41)	0.93	0.95
	2.65 (1.65‒5.16)	2.76 (1.65‒5.73)	−0.08 (−0.51, 0.35)	0.73	0.87
IFNγ	64.3	71.7			
	17.11 ± 20.31	13.39 ± 27.62	0.02 (−0.44, 0.48)	0.93	0.95
	4.99 (2.65‒9.10)	4.67 (2.65‒12.23)	0.07 (−0.39, 0.54)	0.75	0.87
CXCL9	97.6	98.9			
	387.41 ± 493.86	858.85 ± 4699.59	−0.15 (−0.51, 0.21)	0.41	0.80
	263.85 (152.26‒367.29	197.44 (120.38‒386.31)	−0.16 (−0.50, 0.19)	0.37	0.70
CXCL10	100.00	100.00			
	351.98 ± 312.54	356.93 ± 393.85	−0.03 (−0.28, 0.21)	0.78	0.95
	255.81 (208.10‒353.19)	246.76 (178.86‒395.15)	−0.09 (−0.33, 0.15)	0.47	0.79
CCL19	97.6	100.00			
	71.35 ± 55.61	77.92 ± 53.95	−0.19 (−0.44, 0.06)	0.14	0.80
	66.55 (43.54‒85.99)	59.26 (36.98‒77.29)	−0.14 (−0.39, 0.11)	0.26	0.70
Th17					
IL‐17F	11.9	21.7			
	746.00 ± 637.05	198.50 ± 289.89	−5.06 (−13.07, 2.94)	0.22	0.80
	0.01 (0.01‒0.01)	0.01 (0.01‒0.01)	−3.88 (−11.58, 3.82)	0.32	0.70
IL‐21	57.1	53.3			
	6.19 ± 5.05	14.25 ± 28.77	−0.22 (−0.71, 0.26)	0.37	0.80
	3.26 (2.48‒7.13)	2.77 (2.48‒5.45)	−0.29 (−0.78, 0.20)	0.25	0.70
IL‐27	95.2	97.8			
	1628.50 ± 1630.70	1576.78 ± 1141.95	−0.35 (−1.06, 0.37)	0.34	0.80
	1300.0 (960.0‒1815.0)	1220.0 (750.0‒1472.5)	−0.43 (−1.15, 0.29)	0.24	0.70
IL‐23	52.4	48.9			
	1555.52 ± 3441.82	2517.60 ± 5401.18	−0.31 (−1.44, 0.81)	0.59	0.91
	75.14 (75.14‒771.31)	70.45 (75.14‒370.29)	−0.19 (−1.29, 0.90)	0.73	0.87

Abbreviations: CI, confidence interval; IL, interleukin; INF‐γ, interferon γ; LB, lyme borreliosis; TNF‐α, tumor necrosis factor α.

a Data in the first row are the percentage of samples with detectable levels. Data in the second row are mean ± standard deviation for values above the detection limit. Data in the third row are median and interquartile range.

b Differences between patients with LB‐associated constitutional symptoms at enrollment and patients without these symptoms as estimated using the Tobit regression model.

c Differences between patients with LB‐associated constitutional symptoms at enrollment and patients without these symptoms as estimated using the Tobit regression model, and adjusted for sex, age, and dissemination (multiple vs. solitary erythema migrans).

d *p* Value, adjusted for multiple comparisons.

## 4 DISCUSSION

In this study, we evaluated systemic inflammatory immune responses in patients with two clinically distinct manifestations of early infection with *B. afzelii*: early localized and early disseminated LB, manifesting as SEM and MEM. We did not find statistically significant differences in the serum concentrations of 15 inflammatory mediators tested, although there was a trend towards higher levels of CXCL10 and lower levels of CCL19 in patients with MEM. In addition, we did not find differences in systemic inflammatory responses when comparing patients with and without LB‐associated constitutional symptoms. To our knowledge, this is the first study comparing systemic immune responses in patients with SEM and MEM caused by *B. afzelii*. In an earlier study of European patients with SEM, higher serum levels of CXCL9 were found in 45 patients presenting with LB‐associated symptoms than in 41 of those who were asymptomatic, and higher serum levels of CXCL10 were associated with negative borrelial skin culture result.^20^ However, that study excluded samples with undetectable values of inflammatory mediators and lacked multiplicity adjustments.^28^ A similar study did not provide a detailed description on how to address sub‐detectable values of inflammatory mediators.^21^ For samples with concentrations below the lowest standard, Eckman et al.^22^ assigned a midpoint of the interval extending from zero to the lower detection limit. When the proportion of undetectable results is large, as was the case in a previous study analyzing immune responses in European patients with EM^20^ and the present study, cytokine or chemokine values close to zero should not be ignored as they also represent the reality of the immunologic response. Therefore, in this study, we applied a method that included all cytokine or chemokine results and found that the levels of 15 tested serum cytokines or chemokines were not statistically different between patients with SEM and MEM or between patients reporting LB‐associated symptoms and those who were asymptomatic. We also found no association between cytokine or chemokine levels and duration of disease as assessed by duration and diameter of erythema before referral to our outpatient clinic suggesting no significant dynamic variations to systemic immune responses over time. We believe that application of a censored data method, such as the one we used in the present analysis provides a more comprehensive assessment of the weak inflammatory responses found in serum of patients with EM, caused by *B. afzelii*.

Studies comparing immune responses in patients with SEM and MEM caused by *B. burgdorferi* provide inconsistent results.^5^, ^10^, ^12^, ^13^ Salazar et al. who compared immune responses in 15 patients with SEM and 6 patients with MEM caused by *B. burgdorferi*, found significantly lower serum concentrations of IFN‐α, TNF‐α, and IL‐2 in patients with SEM than in those with MEM.^5^ On the other hand, in two other U.S. studies, the frequency of hematogenous dissemination of borreliae, as based on a positive polymerase chain reaction (PCR) result in blood or the presence of MEM, did not correlate with the levels of chemokines or cytokines in serum samples.^10^, ^12^ The latter findings parallel those of another U.S. study analyzing transcriptome, where no difference in gene transcription of a large number of biomarkers in peripheral blood mononuclear cells (PBMC) was found between 17 patients with SEM and 12 patients with MEM.^13^ The divergence of results of these studies could possibly be explained by differences in the patient populations enrolled, for example, defining disseminated *B. burgdorferi* infection by the PCR positivity in blood may have blurred distinctions between the groups of patients with and without disseminated infection, because not all patients with hematogenous dissemination have a positive PCR result in blood.^10^ Moreover, lack of statistical power due to low sample numbers, sampling at different time points along the evolution of EM, or differences in the statistical approach may have contributed to discordant results. Furthermore, results of transcriptome analysis and those of the cytokine or chemokine analysis may not be consistent due to dynamic and differential interactions between RNA and protein kinetics.^29^

In *B. burgdorferi* infection, the association between higher levels of innate and Th1 inflammatory mediators in serum and more symptomatic early infection (more LB‐associated constitutional symptoms) in patients with EM, suggests that innate and adaptive immune responses are important for the pathogenesis of LB‐associated symptoms.^12^, ^21^, ^30^ However, findings have not been consistent across studies. Salazar et al found that out of several cytokines tested (IFN‐α, IFN‐γ, TNF‐α, IL‐2, IL‐4, IL‐6, or IL‐10), only serum IFN‐γ seemed to correlate with symptom scores in patients with SEM or MEM.^5^ Differential expression of an interferon‐dominated transcriptional signature in PBMC over time appeared to track with early disseminated disease and resolution of symptoms in one study.^31^ Conversely, another study found no difference in gene transcription of a large number of biomarkers in PBMC between patients with persistent post‐LD symptoms and those who were asymptomatic at 6 months after having received antibiotic therapy for EM caused by *B. burgdorferi*.^13^

The cytokine or chemokine levels in the serum samples of 12 European patients with EM caused by *B. afzelii* or *B. garinii* were significantly lower from those in patients with EM caused by *B. burgdorferi* and similar to those in normal control subjects, yet half of European patients reported LB‐associated symptoms.^10^ It is uncertain how systemic cytokines or chemokines would decisively mediate symptoms in infection with *B. burgdorferi* in the U.S., but not in European LB. This suggests that serum inflammatory markers may not reflect the principal pathogenetic mechanism by which dissemination of LB, in the form of MEM or LB‐associated constitutional symptoms, occurs, implying other mechanisms at play, such as variations in the inherent hematogenous potential of different *Borreliae* species or strains or local immune response. The latter was suggested recently by Marques et al.,^32^ who assessed the transcriptome of EM skin lesions in patients infected with *B. burgdorferi*. The transcriptome assessment of EM skin lesions revealed that interferon‐inducible genes coding for enzymes involved in tryptophan metabolism may mediate localized immunosuppressive or tolerogenic host responses to infection, suggesting a mechanism facilitating spirochetal dissemination.^32^ Induced genes involved innate immune responses, cell migration and chemotaxis, and microbial defense responses.^32^ It is possible that a dampening of the net inflammatory response may be permissive of borrelial immune evasion and allow dissemination. In a mouse model, IL‐10 deficiency was associated with more inflammation and increased borrelial clearance.^33^ An in vitro study using mouse macrophages and dendritic cells revealed that these cells produced IL‐10 in response to *B. burgdorferi* thereby blocking certain critical antigen presenting cell functions including secretion of inflammatory cytokines or chemokines. The resulting IL‐10 mediated anti‐inflammatory immune response was hypothesized to have a major influence on dissemination and persistence of *B. burgdorferi*.^34^ Despite this intriguing concept based on in vitro and animal studies, our results did not reveal a significant difference in serum IL‐10 between patients with SEM and MEM.

Development of specific *B. burgdorferi* immunoglobulins has been correlated with increased spirochetal clearance and even prevented establishment of borrelial infection in mouse models.^35^, ^36^, ^37^, ^38^ In patients with EM, the levels of inflammatory mediators, particularly TH17‐associated cytokines, correlate directly with *B. burgdorferi* immunoglobulin G antibodies, suggesting a beneficial role for these responses in control of early infection.^21^ Although we found a trend towards increased anti‐inflammatory cytokine IL‐10 in patients with specific borrelial IgG, these associations were not significant. Perhaps a more adequately powered study might shed light on the relationship between anti‐ and proinflammatory cytokines and specific borrelial IgG.

## 5 LIMITATIONS

Our study has several limitations. The major limitation of our study is that inflammatory mediators were only assessed in the sera of patients with EM, yet assessing local immune responses in skin samples from EM lesions and measuring other biomarkers might provide more relevant information in regard to the pathogenesis of borrelial dissemination in the form of MEM and LB‐associated symptoms. Second, we did not include a control group of healthy individuals without LB or other acute infection to set the baseline for inflammatory mediators. However, we believe this is not a major drawback because the analysis aimed to explore potential differences in inflammatory responses in patients with clinically distinct LB manifestations regardless of the absolute inflammatory mediator concentration values. Third, sampling was performed at specific time points, rather than according to symptoms. Conceivably, a more individualized testing scheme may be more appropriate. Finally, the sample size, although being substantially larger than in similar studies, was small for the type of analysis conducted and the number of tested hypotheses, resulting in a potentially underpowered study.

## 6 CONCLUSION

In summary, systemic immune responses represented by selected cytokines or chemokines in serum samples of patients with EM infected with *B. afzelii* do not appear to reveal a significant mechanism regulating dissemination of early infection in the form of MEM or LB‐associated symptoms. Future studies might focus on local immune responses to further elucidate the pathogenesis of these phenomena.

## CONFLICT OF INTERESTS

The authors declare that there are no conflict of interest.

## AUTHOR CONTRIBUTIONS

Daša Stupica designed the study. Tjaša Cerar Kišek and Eva Ružić‐Sabljić conducted the laboratory experiments. Tjaša Cerar Kišek and Daša Stupica conducted data gathering. R.B. performed the statistical analyses. Daša Stupica, Stefan Collinet‐Adler, and Fajko F. Bajrović conducted data interpretation, searched the literature and wrote the manuscript. All the authors approved the article to be submitted.

## ETHICS STATEMENT

The study was approved by the Medical Ethics Committee of the Ministry of Health of the Republic of Slovenia (No. 0120/‐670/2017/7) and registered at Clinical Trials registration, NCT03980015. All patients gave written consent to participate.

## Data Availability

The data that support the findings of this study are available from the corresponding author upon reasonable request.

## References

[1] Stanek G , Wormser GP , Gray J , Strle F . Lyme borreliosis. Lancet. 2012;379:461‐473. 21903253 10.1016/S0140-6736(11)60103-7

[2] Steere AC , Strle F , Wormser GP , et al. Lyme borreliosis. Nat Rev Dis Prim. 2016;2:16090. 10.1038/nrdp.2016.90 27976670 PMC5539539

[3] Melski JW , Reed KD , Mitchell PD , Barth GD . Primary and secondary erythema migrans in Central Wisconsin. Arch Dermatol. 1993;129:709‐716. 8389536

[4] Wormser GP , McKenna D , Carlin J , et al. Brief communication: hematogenous dissemination in early lyme disease. Ann Intern Med. 2005;142:751‐755. 15867407 10.7326/0003-4819-142-9-200505030-00011

[5] Salazar JC , Pope CD , Sellati TJ , et al. Coevolution of markers of innate and adaptive immunity in skin and peripheral blood of patients with erythema migrans. J Immunol. 2003;171:1660‐1670. 10.4049/jimmunol.171.5.2660 12928420

[6] Schuijt TJ , Hovius JWR , Van Burgel ND , et al. The tick salivary protein Salp15 inhibits the killing of serum‐sensitive Borrelia burgdorferi sensu lato isolates. Infect Immun. 2008;76:2888‐2894. 18426890 10.1128/IAI.00232-08 PMC2446733

[7] Strle F . Comparison of culture‐confirmed erythema migrans caused by Borrelia burgdorferi sensu stricto in New York State and by Borrelia afzelii in Slovenia. Ann Intern Med. 1999;130:32‐36. 9890847 10.7326/0003-4819-130-1-199901050-00006

[8] Cerar T , Strle F , Stupica D , et al. Differences in genotype, clinical features, and inflammatory potential of *Borrelia burgdorferi* sensu stricto strains from Europe and the United States. Emerg Inf Dis. 2016;22:818‐827. 10.3201/eid2205.151806 PMC4861522 27088349

[9] Jones KL , Muellegger RR , Means TK , et al. Higher mRNA levels of chemokines and cytokines associated with macrophage activation in erythema migrans skin lesions in patients from the United States than in patients from Austria with Lyme borreliosis. Clin Infect Dis. 2008;46:85‐92. 18171218 10.1086/524022

[10] Strle K , Drouin EE , Shen S , et al. *Borrelia burgdorferi* stimulates macrophages to secrete higher levels of cytokines and chemokines than *Borrelia afzelii* or *Borrelia garinii* . J Infect Dis. 2009;200:1936‐1943. 19909078 10.1086/648091 PMC2783242

[11] Stupica D , Maraspin V , Bogovič P , et al. Comparison of clinical course and treatment outcome for patients with early disseminated or early localized lyme borreliosis. JAMA Dermatology. 2018;154:1050‐1056. 30073319 10.1001/jamadermatol.2018.2306 PMC6143036

[12] Strle K , Jones KL , Drouin EE , Li X , Steere AC . *Borrelia burgdorferi* RST1 (OspC type A) genotype is associated with greater inflammation and more severe Lyme disease. Am J Pathol. 2011;178:2726‐2739. 21641395 10.1016/j.ajpath.2011.02.018 PMC3123987

[13] Bouquet J , Soloski MJ , Swei A , et al. Longitudinal transcriptome analysis reveals a sustained differential gene expression signature in patients treated for acute Lyme disease. mBio. 2016;7:1‐11. 10.1128/mBio.00100-16 PMC4791844 26873097

[14] Stupica D , Velušček M , Blagus R , et al. Oral doxycycline versus intravenous ceftriaxone for treatment of multiple erythema migrans: an open‐label alternate‐treatment observational trial. J Antimicrob Chemother. 2018;73:1352‐1358. 29385444 10.1093/jac/dkx534

[15] Cerar D , Cerar T , Ružić‐Sabljić E , Wormser PG , Strle F . Subjective symptoms after treatment of early Lyme disease. Am J Med. 2010;123:79‐86. 20102996 10.1016/j.amjmed.2009.05.011

[16] Stupica D , Lusa L , Maraspin V , et al. Correlation of culture positivity, PCR positivity, and burden of borrelia burgdorferi Sensu Lato in Skin Samples of erythema migrans patients with clinical findings. PLOS One. 2015;10:1‐10. 10.1371/journal.pone.0136600 PMC4564201 26352832

[17] Stupica D , Lusa L , Ružić‐Sabljić E , Cerar T , Strle F . Treatment of erythema migrans with doxycycline for 10 days versus 15 days. Clin Infect Dis. 2012;55:343‐350. 22523260 10.1093/cid/cis402

[18] Stanek G , Fingerle V , Hunfeld KP , et al. Lyme borreliosis: clinical case definitions for diagnosis and management in Europe. Clin Microbiol Infect. 2011;17:69‐79. 20132258 10.1111/j.1469-0691.2010.03175.x

[19] Wormser GP , Dattwyler RJ , Shapiro ED , et al. The clinical assessment, treatment, and prevention of Lyme disease, human granulocytic anaplasmosis, and babesiosis: clinical practice guidelines by the Infectious Diseases Society of America. Clin Infect Dis. 2006;43:1089‐1134. 17029130 10.1086/508667

[20] Strle K , Stupica D , Drouin EE , Steere AC , Strle F . Elevated levels of IL‐23 in a subset of patients with post–lyme disease symptoms following erythema migrans. Clin Inf Dis. 2014;58:372‐380. 10.1093/cid/cit735 PMC3890340 24218102

[21] Strle K , Sulka KB , Pianta A , et al. T‐Helper 17 cell cytokine responses in lyme disease correlate with Borrelia burgdorferi antibodies during early infection and with autoantibodies late in the illness in patients with antibiotic‐refractory Lyme arthritis. Clin Infect Dis. 2017;64:930‐938. 28077518 10.1093/cid/cix002 PMC5850331

[22] Eckman EA , Pacheco‐Quinto J , Herdt AR , Halperin JJ . Neuroimmunomodulators in neuroborreliosis and lyme encephalopathy. Clin Infect Dis. 2018;67:80‐88. 29340592 10.1093/cid/ciy019

[23] Ružić‐Sabljić E , Zore A , Strle F . Characterization of *Borrelia burgdorferi* sensu lato isolates by pulsed‐field gel electrophoresis after MluI restriction of genomic DNA. J Clin Microbiol. 2008;159:441‐448. 10.1016/j.resmic.2008.05.005 18586084

[24] Postic D , Assous MV , Grimont PAD , Baranton G . Diversity of *Borrelia burgdorferi* sensu lato evidenced by restriction fragment length polymorphism of rrf (5S)‐rrl (23S) intergenic spacer amplicons. Int J Syst Bacteriol. 1994;44:743‐752. 7981102 10.1099/00207713-44-4-743

[25] Tobin J . Estimation of relationships for limited dependent variables. Econometrica. 1958;26:24‐36.

[26] Best DJ , Roberts DE . The upper tail probabilities of Spearman's Rho. Appl Stat. 1975;24:377‐379.

[27] R Development Core Team . R: a language and environment for statistical computing. R foundation for statistical computing. Vienna. 2016. http://www.R-project.org

[28] Parvu M , Parvu V . Statistical issues when searching for predictors of post‐Lyme disease symptoms. Clin Infect Dis. 2014;58:1199‐1200. 24470274 10.1093/cid/ciu059

[29] Hoogendijk AJ , Pourfarzad F , Aarts CEM , et al. Dynamic transcriptome‐proteome correlation networks reveal human myeloid differentiation and veutrophil‐specific programming. Cell Rep. 2019;29:2505‐2519. 31747616 10.1016/j.celrep.2019.10.082

[30] Müllegger RR , McHugh G , Ruthazer R , Binder B , Kerl H , Steere AC . Differential expression of cytokine mRNA in skin specimens from patients with erythema migrans or acrodermatitis chronica atrophicans. J Invest Dermatol. 2000;115:1115‐1123. 11121150 10.1046/j.1523-1747.2000.00198.x

[31] Petzke MM , Volyanskyy K , Mao Y , et al. Global transcriptome analysis identifies a diagnostic signature for early disseminated Lyme disease and its resolution. mBio. 2020;11:1‐15. 10.1128/mBio.00047-20 PMC7078463 32184234

[32] Marques A , Schwartz I , Wormser GP , et al. Transcriptome assessment of erythema migrans skin lesions in patients with early Lyme disease reveals predominant interferon signaling. J Infect Dis. 2018;217:158‐167. 10.1093/infdis/jix563 PMC5853807 29099929

[33] Lazarus JJ , Meadows MJ , Lintner RE , Wooten RM . IL‐10 deficiency promotes increased Borrelia burgdorferi clearance predominantly through enhanced innate immune responses. J Immunol. 2006;177:7076‐7085. 17082624 10.4049/jimmunol.177.10.7076

[34] Chung Y , Zhang N , Wooten RM . Borrelia burgdorferi elicited‐IL‐10 suppresses the production of inflammatory mediators, phagocytosis, and expression of co‐stimulatory receptors by murine macrophages and/or dendritic cells. PLOS One. 2013;8:1‐13. 10.1371/journal.pone.0084980 PMC3868605 24367705

[35] Radolf JD , Caimano MJ , Stevenson B , Hu TL . Of ticks, mice, and Men. Nat Rev Microbiol. 2012;10:87‐99. 22230951 10.1038/nrmicro2714 PMC3313462

[36] Mbow ML , Gilmore RD , Titus RG . An OspC‐specific monoclonal antibody passively protects mice from tick‐transmitted infection by borrelia burgdorferi B31. Infect Immun. 1999;67:5470‐5472. 10496931 10.1128/iai.67.10.5470-5472.1999 PMC96906

[37] Barthold SW , Hodzic E , Tunev S , Feng S . Antibody‐mediated disease remission in the mouse model of lyme borreliosis. Infect Immun. 2006;74:4817‐4825. 16861670 10.1128/IAI.00469-06 PMC1539599

[38] McKisic MD , Barthold SW . T‐cell‐independent responses to borrelia burgdorferi are critical for protective immunity and resolution of lyme disease. Infect Immun. 2000;68:5190‐5197. 10948143 10.1128/iai.68.9.5190-5197.2000 PMC101777

